# Clinical profile of rural community hospital inpatients in Sweden – a register study

**DOI:** 10.1080/02813432.2021.1882086

**Published:** 2021-02-11

**Authors:** Mante Hedman, Kurt Boman, Margareta Brännström, Patrik Wennberg

**Affiliations:** a Department of Public Health and Clinical Medicine, Umeå University, Umeå, Sweden; b Department of Nursing, Skellefteå Campus, Umeå University, Umeå, Sweden

**Keywords:** Rural health service, health services research, hospital, rural, inpatient, health services for the aged, geriatrics

## Abstract

**Objective:**

Patients in Sweden’s rural community hospitals have not been clinically characterised. We compared characteristics of patients in general practitioner-led community hospitals in northern Sweden with those admitted to general hospitals.

**Design:**

Retrospective register study.

**Setting:**

Community and general hospitals in Västerbotten and Norrbotten counties, Sweden.

**Patients:**

Patients enrolled at community hospitals and hospitalised in community and general hospitals between 1 January 2010 and 31 December 2014.

**Outcome measures:**

Age, sex, number of admissions, main, secondary and total number of diagnoses.

**Results:**

We recorded 16,133 admissions to community hospitals and 60,704 admissions to general hospitals. Mean age was 76.8 and 61.2 years for community and general hospital patients (*p* < .001). Women were more likely than men to be admitted to a community hospital after age adjustment (odds ratio (OR): 1.11; 95% confidence interval (CI): 1.09–1.17). The most common diagnoses in community hospital were heart failure (6%) and pneumonia (5%). Patients with these diagnoses were more likely to be admitted to a community than a general hospital (OR: 2.36; 95% CI: 2.15–2.59; vs. OR: 3.32: 95% CI: 2.77–3.98, respectively, adjusted for age and sex). In both community and general hospitals, doctors assigned more diagnoses to men than to women (both *p*<.001).

**Conclusions:**

Patients at community hospitals were predominantly older and women, while men were assigned more diagnoses. The most common diagnoses were heart failure and pneumonia. Our observed differences should be further explored to define the optimal care for patients in community and general hospitals.

**Key points**
The patient characteristics at Swedish general practitioner-led rural community hospitals have not yet been reported. This study characterises inpatients in community hospitals compared to those referred to general hospitals.
  • Patients at community hospitals were predominantly older, with various medical conditions that would have led to a referral to general hospitals elsewhere in Sweden.
  • Compared to men, women were more likely to be admitted to community hospitals than to general hospitals, even after adjustment for age. To the best of our knowledge, this pattern has not been reported in other countries with community hospitals.
  • In both community hospitals and general hospitals, doctors assigned more diagnoses to men than to women.

## Introduction

General practitioner (GP)-led community hospitals provide health care mainly in sparsely populated rural areas, including in northern Sweden. Community hospitals are pragmatic solutions adapted to local health care needs [1,2], without a specific definition. An international definition applicable for a Swedish context is a hospital where the admission, care and discharge of patients are under the direct control of a GP ‘who is paid for this service through a bed fund, or its equivalent’ [3].

In Sweden, primary health care is mainly organised into primary care units (PCUs) rather than GP-owned practices. PCUs can be private or publicly owned by the county councils. These units are responsible for local primary health care provided by employed GPs, registered nurses, district nurses, physiotherapists, occupational therapists, psychologists and midwives, among others. In Norrbotten and Västerbotten counties in northern Sweden, 14 community hospitals are scattered in rural municipalities covering almost a quarter of the area of Sweden. These community hospitals are the local PCUs, and inhabitants in these municipalities are enrolled at the community hospital. Compared to usual Swedish PCUs, the community hospitals offer a wider range of services, including a hospital ward, plain X-ray and emergency rooms. In community hospital wards, GPs observe and treat a variety of acute conditions, offer post-acute care for patients after treatment at a general hospital, and provide end-of-life care.

In contrast to general hospital wards, community hospital wards are not confined to one medical specialty but deal with different medical conditions. A group that reviewed practices in the United Kingdom (UK) concluded that community hospitals contribute to ‘acute, terminal, and elderly care, as well as respite care and rehabilitation services’ [4]. In another study, community hospitals provided acute medical care for a wide range of patients, and they were suggested to mainly substitute for general hospital care [5]. Several studies have shown that community hospital admissions are especially suitable for multimorbid elderly patients [6–9]. The perspective differs considerably between a hospital specialist in a general hospital ward, with a deep competence in a narrow discipline, and a specialist in general medicine (a Swedish GP) in a community hospital ward, with a broader and less specialised medical competence and an expected holistic view of the patient. Moffat and Mercer concluded that the management of multimorbidity involves many challenges, requiring a holistic approach by a generalist [10].

We believe that it is important that hospitalised patients are admitted to an optimal level of care, with respect to patient safety and convenience as well as cost efficiency. In Sweden, the rural community hospitals represent an intermediate level of hospital care between the general hospital level and the municipal nursing homes. Equivalents to the Swedish community hospital model have been studied in other countries but not in Sweden. Consequently, patients in rural community hospitals in Sweden have not been characterised from a clinical perspective. Therefore, we aimed to characterise patients admitted to hospitals in Norrbotten and Västerbotten counties and to compare hospitalisations at community hospitals and general hospitals. We hypothesised that patients admitted to community hospitals differ from those admitted to general hospitals with regard to age, sex and main diagnoses.

## Materials and methods

### Geographical context

Sweden is sparsely populated, with a density of 24 people/km^2^, compared to 120/km^2^ in the EU. Municipalities with community hospitals have extremely sparse populations (0.78/km^2^). In these municipalities, the median age was 49 years in 2014, compared to 41 years in Sweden nationally (computed from data in Statistics Sweden [11]) and 42 years in the EU [12]. The areas supported by community hospitals in the present study are described in Table A1.

### Study population and data source

Patients enrolled at community hospitals in Västerbotten and Norrbotten counties were included if they were hospitalised at community hospitals or other hospitals within their county between 1 January 2010 and 31 December 2014. Hospital registry data from the county councils of Västerbotten and Norrbotten were retrieved for all hospitalisations during this period and included in the study. Variables were age at admission, sex, patient’s chosen PCU/community hospital, treating hospital clinic, date of admission, length of stay (LOS), main diagnosis, the first five secondary diagnoses and total number of diagnoses during the hospitalisation.

Diagnoses were coded using the ICD-10 (International Statistical Classification of Diseases and Related Health Problems 10th Revision) system [13]. ICD-10 codes were reduced to the first three characters. All diagnoses with extensions (e.g. J18.1, J18.2, J18.8 and J18.9) were collected into the same diagnosis code (J18). Different dementia (main) diagnoses (F00, F01, F02, F03 and F05.1) were aggregated into one dementia diagnosis.

### Statistical analysis

Data are presented in frequency tables with mean (standard deviation (SD)) and/or median and quartiles (min/max). Secondary diagnoses 1–5 were aggregated to calculate the total number of admissions with each secondary diagnosis. Additional diagnoses after the main diagnosis and the first five secondary diagnoses were not classified in the data. Normally distributed continuous variables were tested using the independent two-sample *t*-test. Categorical variables were tested using the *χ*^2^ test. Significance was set at *p* < .05. Odds ratios (ORs) for admission to community hospitals compared to general hospitals were estimated using logistic regression. Binary and multivariable logistic regression was used. Association of admittance to community hospitals was adjusted for age or sex. Association for separate diagnoses for admission to community hospitals was adjusted for age (model 1) and for age and sex (model 2).

### Ethics

This study was approved by the Research Ethics Committee in Umeå (ref no. 2016/52-31Ö).

## Results

For the investigated variables, there were no missing values. The total number of admissions to general hospitals and community hospitals in the period 1 January 2010 through 31 December 2014 was 76,837 (Table 1). Patients were admitted to 174 different hospital wards: 74 in Norrbotten and 100 in Västerbotten. These wards included 14 community hospital wards and hospital wards at eight general hospitals (five in Norrbotten and three in Västerbotten). Of a total 16,131 admissions to community hospitals, 9900 were in Norrbotten and 6231 in Västerbotten.

**Table 1.** Characteristics of patients in community hospitals and general hospitals.

		Community hospitals			General hospitals		
		Women	Men^a^	All	Women	Men^a^	All^b^
Number of admissions		8540	7593***	16,133	30,276	30,428^ns^	60,704
Number of patients		3734	3309***	7043	11,531	10,193***	21,724
Number of adm/patient, mean (SD)		2.29 (2.32)	2.29 (2.19)		2.63 (2.92)	2.99 (3.30)***	
Mean age, years (SD)		78.0 (13.7)	75.4 (13.8)***	76.8 (13.8)	60.1 (23.2)	62.4 (20.9)***	61.2 (22.1)***
Age	25%	74	70	72	43	55	50
Quartiles	50%	81	78	80	67	68	68
Years	75%	87	85	86	78	77	78
Length of stay, days, mean (SD)		5.62 (6.79)	5.30 (6.49)**	5.47 (6.65)	4.30 (7.43)	4.68 (13.1)***	4.49 (10.7)***
Number of diagnoses		2.22 (1.65)	2.36 (1.80)***	2.29 (1.72)	3.00 (2.15)	3.25 (2.35)***	3.13 (2.25)***
Mean (SD)							
Number of diagnoses		2 (0–16)	2 (0–16)	2 (0–16)	2 (1–29)	3 (0–25)	3 (0–29)
Median (min/max)							

^a^ Differences between men and women: **p < .01, ***p < .001.

^b^ Differences between community hospitals and general hospitals (all patients): ***p < .001.

**Figure 1.**
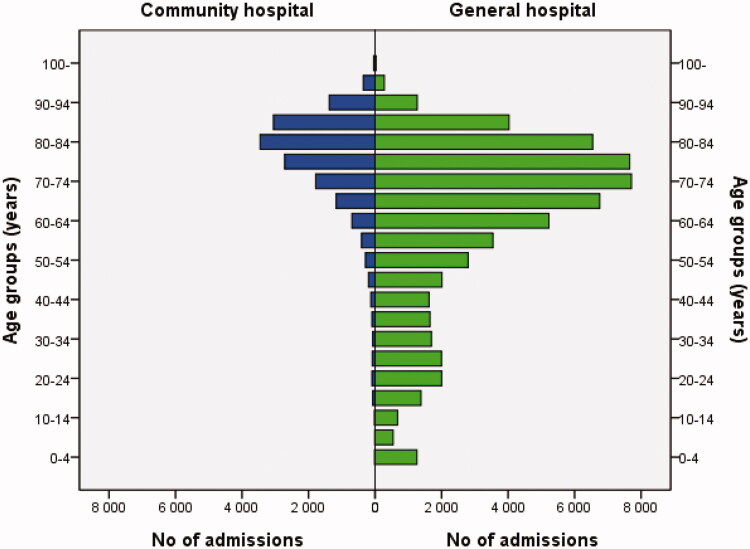
Histogram comparing age distribution between community hospitals and general hospitals presented in 5-year age intervals.

Patient characteristics are shown in Table 1. The mean age of community hospital patients was higher than that of general hospital patients, years (SD): 76.8 (13.8) vs. 61.2 (22.1), *p* < .001. LOS was longer in community hospitals than in general hospitals (*p* < .001). Men were assigned more diagnoses at discharge than women in both community hospitals and general hospitals (both *p* < .001). Differences in age distributions are illustrated in Figure 1. Women had higher ORs for being admitted to community hospitals compared to men. This difference remained significant after adjustment for age (Table 2). As community hospitals in northern Sweden do not accommodate childbirth, calculations were also performed in patients >50 years, to exclude childbirths in general hospitals. With this exclusion, the OR was still higher for women to be admitted to community hospitals (Table 2).

**Table 2.** Odds ratios for admission to a community hospital compared to a general hospital calculated for sex and age^a^.

	Crude	Adjusted
All ages		
Sex (woman vs. man)	1.13 (1.09–1.17)	1.11 (1.07–1.15)^b^
Age	1.06 (1.05–1.06)	1.06 (1.05–1.06)^c^
Age >50 years		
Sex (woman vs. man)	1.30 (1.25–1.35)	1.10 (1.06–1.15)^b^
Age	1.07 (1.069–1.073)	1.07 (1.068–1.072)^c^

^a^ Odds ratios (95% confidence intervals) for age represent the increase in probability for admission to a community hospital for each year of increase in age. Example: for age > 50 years, crude OR (95% CI) for 5 and 10 years increase in age are 1.40 (1.39–1.41) and 1.89 (1.85–1.93), respectively.

^b^ OR (95% CI) for sex (woman), adjusted for age.

^c^ OR (95% CI) for age, adjusted for sex.

A total of 211 different main diagnoses were recorded at community hospitals and 217 at general hospitals. The most common main diagnoses in community hospitals were heart failure (6%) and pneumonia (5%). Table 3 lists the 10 most frequent main diagnoses in community hospitals and general hospitals, with numbers and percentages, and in Table 4, those in community hospitals are included in a multivariable model. Patients with these diagnoses, except for atrial fibrillation/flutter, were more likely to be admitted to a community hospital than a general hospital.

**Table 3.** Distribution of the 10 most common main diagnoses from hospitalisations in community hospitals and general hospitals, 2010–2014.

Community hospital				General hospital			
Main diagnosis	ICD-10	Fq	%	Main diagnosis	ICD-10	Fq	%
Heart failure	I50	965	6.0	Encounter for other postprocedural aftercare	Z48	3863	6.4
Pneumonia	J18	800	5.0	Acute myocardial infarction	I21	2262	3.7
Atrial fibrillation and flutter	I48	401	2.5	Encounter for full-term uncomplicated delivery	O80	1901	3.1
Abdominal and pelvic pain	R10	399	2.5	Pain in throat and chest	R07	1280	2.1
Type 2 diabetes mellitus	E11	359	2.2	Fracture of femur	S72	1218	2.0
Urinary tract infection	N30	321	2.0	Atrial fibrillation and flutter	I48	1214	2.0
Orthopaedic aftercare	Z47	313	1.9	Angina pectoris	I20	1142	1.9
Chronic obstructive pulmonary disease	J44	308	1.9	Cerebral infarction	I63	1112	1.8
Dizziness and giddiness	R42	307	1.9	Abdominal and pelvic pain	R10	987	1.6
Dementia diagnoses	F00, F01, F02, F03, F05.1	382	1.9	Heart failure	I50	982	1.6
Total		4475	27.7	Total		15,961	26.2

**Table 4.** OR (95% confidence intervals) for patients to be admitted to a community hospital vs. community hospital according to the most frequent main diagnoses found in community hospitals^a^.

Main diagnosis (ICD-10)	%	Crude OR	Model 1	Model 2
Heart failure (I50)	6.0	3.87 (3.53–4.24)	2.32 (2.11–2.55)	2.36 (2.15–2.59)
Pneumonia (J18)	5.0	3.97 (3.59–4.38)	3.31 (2.76–3.97)	3.32 (2.77–3.98)
Atrial fibrillation and flutter (I48)	2.5	1.25 (1.11–1.40)	1.02 (0.90–1.14)ns	1.01 (0.90–1.14)ns
Abdominal and pelvic pain (R10)	2.5	1.53 (1.36–1.73)	2.77 (2.42–3.17)	2.73 (2.39–3.13)
Type 2 diabetes mellitus (E11)	2.2	6.65 (5.60–7.90)	5.81 (4.86–6.95)	5.82 (4.87–6.97)
Urinary tract infection (N30)	2.0	43.99 (29.89–64.76)	38.17 (24.99–58.32)	38.13 (24.96–58.25)
Orthopaedic aftercare (Z47)	1.9	5.70 (4.78–6.79)	4.56 (3.77–5.50)	4.52 (3.74–5.46)
Chronic obstructive pulmonary disease (J44)	1.9	3.31 (2.84–3.86)	2.57 (2.20–3.01)	2.55 (2.18–2.99)
Dizziness and giddiness (R42)	1.9	2.93 (2.52–3.41)	2.29 (1.96–2.68)	2.28 (1.95–2.67)
Constipation (K59)	1.9	4.04 (3.42–4.77)	3.31 (2.76–3.97)	3.32 (2.77–3.98)
Dementia diagnosis (F00, F01, F02, F03, F05.1)	1.9	7.31 (6.03–8.87)	4.34 (3.57–5.29)	4.37 (3.59–5.32)

Data are given as percentage of all main diagnoses. Model 1 was adjusted for age. Model 2 was adjusted for age and sex. The variable ‘sex’ and each of the different diagnoses are dichotomised (being a woman or not, having the diagnosis or not), whereas OR calculated for age represents the increase in probability for admission to a community hospital for each year of increase in age.

^a^ All *p* < .001, unless marked ‘ns’ (not significant).

The incidence of admission with a main diagnosis of urinary tract infection (UTI) was 219/100,000 inhabitants, with disproportionate differences between Norrbotten (819/100,000) and Västerbotten (132/100,000). The counties did not differ for the other diagnoses studied.

The most common secondary diagnoses in community hospitals and general hospitals are listed in Table A2. Of 226,687 diagnoses, a total of 48,199 (21%) were not classified because they were in addition to the main diagnosis and the first five secondary diagnoses. Doctors assigned more diagnoses to men than to women in both community hospitals and general hospitals (Table 1).

## Discussion

The main finding of this study was that community hospitals in Sweden predominantly treat the oldest patients for diagnoses associated with older age. A new finding was that when we compared admissions between community hospitals and general hospitals, women had higher ORs for being admitted to community hospitals compared to men, even after adjustment for age. Doctors assigned more diagnoses to men than to women at both community hospitals and general hospitals.

### Strengths and limitations

The strength of this study is that data from all registered hospitalisations were assessed covering 5 years for a defined population. Furthermore, almost every community hospital in Sweden was studied, so in the Swedish medical system, these results would be considered exhaustive. However, community hospitals in other regions of the world have been developed in response to local needs and health care systems, and thus are heterogeneous within and among different countries. Thus, our findings are not generalisable and regionality should be considered when comparing results from different community hospital studies.

For practical reasons, we collected the first six diagnoses registered (main diagnosis and five secondary diagnoses) for all admissions, representing 79% of the total diagnoses. These diagnoses were considered the most important from a medical perspective.

We note the difference in the number of diagnoses between general hospitals and community hospitals, with more diagnoses in general hospitals. Chronic diseases tend to accumulate with age, and community hospital patients are older than general hospital patients. General hospitals offer a larger diversity of medical procedures, which could partly explain the difference. Diagnosis registration behaviour also seemed to differ between GPs and hospital doctors, but we found no other relevant studies for comparison. A medical secretary who reviews all medical records for admissions to community hospitals in Västerbotten informed us that from the perspective of general hospital medical records, GPs document differently from hospital doctors, with fewer details and fewer diagnoses (personal communication). Thus, the difference could trace in part to a practice of community hospital doctors to document in a primary care context, with the intent of being their own readers, rather than using the more structured approach that hospital clinicians follow with an expectation of shared notes. The bottom line is that differences in the number of diagnoses between community hospitals and general hospitals in our study are likely to be explained by other factors and may not be reliable for describing patient multimorbidity.

In this register study, comparisons of LOS between community hospitals and general hospitals should be interpreted with caution. We have insufficient data on referrals from community hospitals to general hospitals and vice versa. Case-mixing occurred to an unknown extent because patients could be treated at both community hospitals and general hospitals or at two different general hospitals in a continuous hospitalisation. The most obvious result from such case-mixing would be that general hospital LOS would be shortened if patients were referred to community hospitals, and vice versa.

### Age and sex perspectives

Community hospitals treated predominantly older patients, in accordance with international findings [6–9]. These patients are more often frail and multimorbid, and the association between older age in a population and use of hospital care is well established. However, patient age does not seem to fully explain the observed increase in emergency admissions for older people [14]. Multimorbidity calls for a holistic perspective in care [10], and we suggest that the generalist perspective of GP doctors and staff in community hospital wards who are treating patients with various conditions could be beneficial for these patient groups.

In 2018, life expectancy was 84.1 years for Swedish women, compared to 80.6 years for Swedish men [15]. Consequently, the oldest age groups have more female hospital patients [16], which is in accordance with the present study’s findings. However, the OR remained higher for women to be admitted to community hospitals rather than general hospitals after adjustment for age, and when selecting all admissions of patients over age 50 years, which excluded obstetric patients. We found no other studies that address these differences in the community hospital model of care.

In contrast, men had higher ORs for admission to general hospitals compared to women. Authors of a review of nursing home residents [17] and a study of the general population of a region in the UK [18] found that men have more frequent hospitalisations than women. These earlier results imply that our finding may reflect a common phenomenon rather than a local behaviour.

The present study also showed that men were assigned more diagnoses than women in both community hospitals and general hospitals, which also is in accordance with national data [19]. From our register data, we cannot explain these differences in hospital admissions and diagnoses between the sexes. Patient factors, such as sex differences (based on biological factors) and gender differences (based on behaviour, lifestyle and life experience), may underlie the variations, as could attitudes and behaviours in the health care organisation. There are many examples in disease management of women receiving different and often unfavourable treatment compared to men [20]. It has been claimed that this disparity is simply the result of physician behaviours [21].

### Diagnoses

The most frequent main diagnoses for patients in community hospitals were diseases of the elderly. Heart failure and pneumonia were the most common in community hospitals but did not rank high at general hospitals in this cohort. Patients with heart failure, pneumonia and most of the other common main diagnoses had high ORs for admission to a community hospital instead of a general hospital. In national comparisons, heart failure was the most common main diagnosis for inpatient care in 2015 [19], and pneumonia was third most common. In areas without community hospitals, most patients with these conditions would probably have been admitted to a general hospital. However, these patient groups seem to be routed to community hospitals rather than general hospitals in the studied areas, implying a divided responsibility in which community hospitals care for elderly patients with diseases most commonly associated with ageing.

In our study, patients with a main diagnosis of dementia were more likely to be admitted to community hospital care than general hospital care. It could be argued that patients with dementia would benefit from hospital care in a community hospital compared to a large hospital (smaller setting, closer to home, holistic view), but we have not yet found evidence for this in the literature.

Urinary tract infection could be debated as a main diagnosis for hospitalisation but has an incidence of 113/100,000 inhabitants in Sweden [16]. We suggest that diffuse symptoms and disability at admission could be explained in part by a UTI at the end of the hospitalisation, resulting in this main diagnosis. These factors also could explain the strong association between UTI and admission to a community hospital because these patient groups require acute supervision and nursing rather than specialised hospital care.

### Comparison with other literature

Our findings suggest that a patient group that is common in hospitals worldwide – elderly patients with chronic diagnoses – are directed to community hospitals, which provide more integrated care, rather than to general hospitals for more general care in rural areas in northernmost Sweden. This is in accordance with a review that summarised studies comparing patient outcomes at community hospitals and general hospitals from medical and health economic perspectives in different countries [2]. As noted, community hospitals seem to provide more integrated care than general hospitals [22,23] to better meet the needs of old patients with multiple chronic conditions. Three Norwegian studies have shown that community hospitals seem to reduce the use of general hospitals with respect to admissions, as well as reducing occupied bed-days [7,24,25]. In Norway municipality, acute bed units were established in municipalities as a result of the Coordination Reform in 2012. These units are intended for short-term stays of patients with conditions manageable by primary care methods, and they fit into the definition of community hospitals. An observational study reports increasing admission rates to a municipality acute bed unit timely coinciding with decreasing admissions to general hospital [26].

In a UK study of 6-month outcomes after acute admission of elderly patients to a community hospital compared to a district general hospital, quality of life and mortality were similar between the two cohorts [27]. Another UK study concluded that local community hospital care is associated with greater independence for older people than is care in wards for geriatric patients in a district general hospital [28]. Furthermore, the cost-effectiveness of post-acute rehabilitation for older people is similar in community hospitals and general hospitals [29]. However, evidence was insufficient in a third UK-based investigation to support that community hospital care reduces acute hospital use or that community hospitals are cost-effective [30].

### Future research

We suggest further studies on the mechanisms underlying sex differences in admissions, treatment and diagnosis, and the community hospital model in Sweden could be a suitable arena for studying these mechanisms in depth. We have initiated qualitative studies of what doctors consider when deciding which patients to keep in the community hospital and which to refer to a general hospital.

We also suggest prospective studies in Sweden to evaluate quality of care and health economics with the community hospital model of care for these patient groups. If such studies show equal or better quality of care and cost efficiency with community hospital compared to general hospital care for the relevant patient groups, this intermediate level of hospital care could be an alternative to the general hospital in urban areas.

## Conclusions

We aimed to characterise patients in GP-led community hospital wards in northern Sweden and compare them to patients admitted to general hospitals. Patients at community hospitals were predominantly older, with various medical conditions that are common in old age and would likely have led to a referral to a general hospital in regions with no community hospitals. Women were more likely to be admitted to community hospitals than general hospitals compared to men, which could not be explained by differences in age distribution between the sexes. In both community hospitals and general hospitals, doctors assigned more diagnoses to men than to women. The mechanisms underlying these differences need further investigation.

## Appendix

**Table A1.** Population, area and population density in municipalities in Västerbotten and Norrbotten, the whole of both counties of the same names, and in Sweden^a^.

	Population, 2014 (*n*)	Area (km^2^)	Population density, 2014 (*n*/km^2^)	Distance to hospital (km)	Distance to hospital (minutes)
*Västerbotten*	262,362	54,672	4.8		
Storuman	5995	7304	0.8	104	77
Vilhelmina	6848	8048	0.9	117	89
Malå	3115	1599	2.0	83	66
Åsele	2838	4224	0.7	88	63
Sorsele	2565	7368	0.4	143	106
Tärnaby (Storuman)	^b^	^b^	^b^	229	166
Dorotea	2757	2 765	1.0	137	97
*Total community hospital area*	24,118	31,308	0.8	129^c^	95^c^
*Norrbotten*	249,987	97,257	2.6		
Pajala	6303	7840	0.8	184	135
Övertorneå	4711	2362	2.0	78	58
Arvidsjaur	6484	5656	1.2	125	93
Jokkmokk	5086	17,614	0.3	94	71
Överkalix	3409	2764	1.2	69	56
Arjeplog	2907	12,556	0.2	210	152
Haparanda	9776	923	10.6	50	35
*Total community hospital area*	38,676	49,715	0.8	116^c^	86^c^
*Sweden*	9,747,355	407,340	23.9		

^a^ Based on statistical data published by Statistiska Centralbyrån. Each municipality is supported by a community hospital.

^b^ Tärnaby is part of Storuman municipality.

^c^ Mean distances. Distance to nearest general hospital from each community hospital is presented as distance in km, and travel time by car was estimated using Google Maps.

**Table A2.** Distribution of the 10 most common secondary diagnoses from hospitalisations in community hospitals and general hospitals, 2010–2014.

Community hospital				General hospital			
Secondary diagnosis	ICD-10	Fq	%	Secondary diagnosis	ICD-10	Fq	%
Essential hypertension	I10	1610	10.0	Essential hypertension	I10	14,609	24.1
Heart failure	I50	1293	8.0	Chronic ischaemic heart disease	I25	6465	10.7
Atrial fibrillation and flutter	I48	1214	7.5	Type 2 diabetes mellitus	E11	5744	9.5
Type 2 diabetes mellitus	E11	1002	6.2	Atrial fibrillation and flutter	I48	5422	8.9
Chronic ischaemic heart disease	I25	761	4.7	Pacemaker *in situ*	Z95	4138	6.8
Unspecified dementia	F03	574	3.6	Heart failure	I50	3254	5.4
Chronic obstructive pulmonary disease	J44	475	2.9	Hyperlipidaemia	E78	3195	5.3
Pneumonia	J18	461	2.9	Angina pectoris	I20	2669	4.4
Disorders of urinary system (cystitis)	N39	452	2.8	Personal history of medical treatment	Z92	1981	3.3
Other anaemias	D64	381	2.4	Chronic obstructive pulmonary disease	J44	1583	2.6
Total		8223	51.0	Total		49,060	80.8

Fq: frequency; ICD-10: The International Statistical Classification of Diseases and Related Health Problems 10th Revision.
